# Preimplantation Genetic Testing for Polygenic Disease Relative Risk Reduction: Evaluation of Genomic Index Performance in 11,883 Adult Sibling Pairs

**DOI:** 10.3390/genes11060648

**Published:** 2020-06-12

**Authors:** Nathan R. Treff, Jennifer Eccles, Diego Marin, Edward Messick, Louis Lello, Jessalyn Gerber, Jia Xu, Laurent C.A.M. Tellier

**Affiliations:** 1 Genomic Prediction Inc. 675 US Highway One, North Brunswick, NJ 08902, USA; jen@genomicprediction.com (J.E.); diego@genomicprediction.com (D.M.); ed@genomicprediction.com (E.M.); lou@genomicprediction.com (L.L.); jia@genomicprediction.com (J.X.); laurent@genomicprediction.com (L.C.A.M.T.); 2 Department of Obstetrics, Gynecology, and Reproductive Sciences, Rutgers University-Robert Wood Johnson Medical School, New Brunswick, NJ 08903, USA; 3 Department of Physics and Astronomy, Hannah Administration Building, Michigan State University, 426 Auditorium Rd., East Lansing, MI 48824, USA; 4 Department of Genetics, Rutgers University, Piscataway, NJ 08854, USA; jag607@dls.rutgers.edu

**Keywords:** preimplantation genetic testing, PGT-P, polygenic risk scoring, genomic index, relative risk reduction

## Abstract

Preimplantation genetic testing for polygenic disease risk (PGT-P) represents a new tool to aid in embryo selection. Previous studies demonstrated the ability to obtain necessary genotypes in the embryo with accuracy equivalent to in adults. When applied to select adult siblings with known type I diabetes status, a reduction in disease incidence of 45–72% compared to random selection was achieved. This study extends analysis to 11,883 sibling pairs to evaluate clinical utility of embryo selection with PGT-P. Results demonstrate simultaneous relative risk reduction of all diseases tested in parallel, which included diabetes, cancer, and heart disease, and indicate applicability beyond patients with a known family history of disease.

## 1. Introduction

In vitro fertilization (IVF) is the most effective treatment for infertility. As clinical and laboratory methods have improved, so has the efficiency of producing blastocysts suitable for intrauterine transfer. As a result, IVF patients and physicians are often faced with determining which specific embryo to transfer. The default strategy for choosing which embryo to transfer involves ranking embryos through careful microscopy-based characterization of development and morphology. However, preimplantation genetic testing (PGT) has become a routine method for embryo selection, now implemented in 40% of all in vitro fertilization (IVF) cycles in the United States [1]. PGT is most commonly applied to select euploid embryos for transfer, while avoiding those embryos designated as aneuploid (PGT-A). The primary objective of PGT-A is to improve the success of IVF in the first attempted embryo transfer [2,3]. Again, the default strategy for choosing which euploid embryo to transfer involves ranking embryos through careful microscopy-based characterization of development and morphology [4].

More recently, the opportunity to characterize the risk of polygenic disease in the preimplantation embryo has been made possible. Polygenic disorders, conditions influenced by genetic variants in multiple genes, account for a large percentage of premature deaths in humans [5,6]. These are largely contributed to by cancer, heart disease, and diabetes. There is a growing body of evidence that the risk of these diseases is higher in individuals seeking fertility treatments [7]. Despite the potential for environmental influence, polygenic disease risk can now be accurately predicted for several common diseases, including cancer, heart disease, and diabetes, using DNA alone [8,9]. We recently demonstrated the ability to achieve equivalent accuracy in genome-wide genotyping of DNA from a preimplantation embryo, as is already achieved when DNA is tested from adults. Therefore, the same performance in predicting polygenic disease in adults can now be achieved in preimplantation embryos [10].

Polygenic risk scoring in adults is often performed and evaluated in the context of entire populations of unrelated people [11]. In contrast, PGT involves evaluating genetic risks among sibling embryos within a single family. This was addressed previously by evaluating blinded DNA from 2601 adult sibling pair families with known type 1 diabetes status. Results demonstrated a 45–72% reduction in the incidence of type 1 diabetes when one sibling was chosen based on a polygenic risk score compared to when one sibling was chosen randomly. This study demonstrated clinical utility of PGT-P in a situation that is similar to PGT for monogenic disease, where intended parents have a known risk of passing on the disease [12].

Independent of fertility, polygenic conditions present in families at a much higher rate compared to monogenic disease, with most polygenic disorders manifesting in adulthood [6]. As such, while it is common for a couple to report a family history of polygenic disorders, it is rarer for a couple to present for PGT-P based on having a previously affected child. Exceptions to this involve polygenic disorders that present with early age of onset, such as type 1 diabetes. In this case, intended parents seeking IVF treatment may already be the parents of an affected child. As reported here, this very case is presented for PGT-P. Still, the spectrum of patients who may consider PGT-P could vary from those being affected or having an affected child to those having an unknown family history of any of the polygenic diseases being tested. In order to address whether PGT-P may apply to intended parents with unknown family history of polygenic disease, several thousand sibling pairs represented in the United Kingdom (UK) Biobank repository were evaluated using a blinded genomic disease index methodology. We also test whether preimplantation embryo genomic index values correlate with the extent of the embryos’ family history. 

## 2. Materials and Methods

### 2.1. PGT-P Case with First Degree Affected Family History

**Figure 1:**
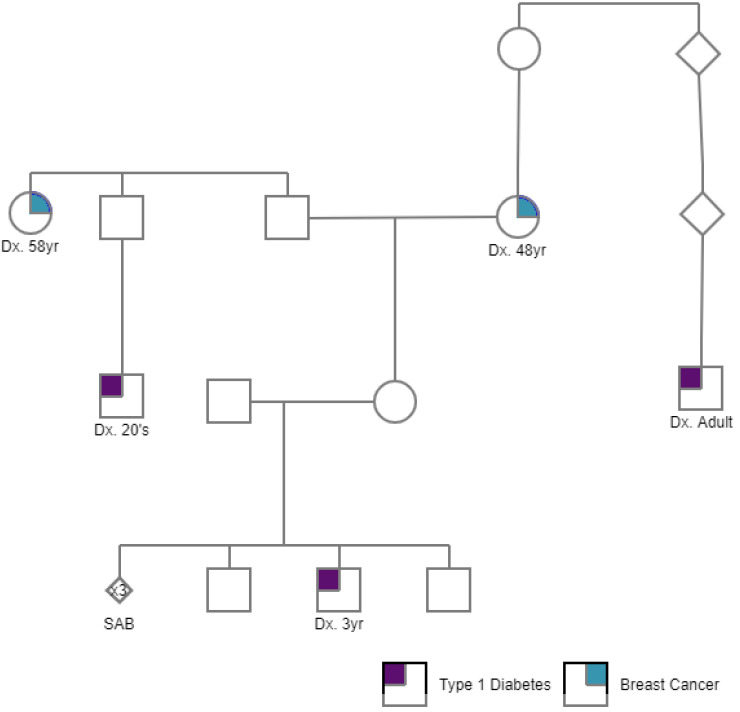
A pedigree of a case presenting for PGT-P with a family history of type 1 diabetes and breast cancer (shown in purple and turquoise, respectively).

A couple with a family history of type 1 diabetes (T1D) presented to the Genomic Prediction Clinical Laboratory and was counseled for and consented to PGT-P as previously described [12]. The couple reported that their 5-year-old son was diagnosed with T1D at 3 years of age, and that two additional relatives, a paternal first cousin and a maternal second cousin, were diagnosed with T1D in their 20s (Figure 1). The patient reported two maternal relatives who were diagnosed with breast cancer. The couple otherwise denied a personal or family history of polygenic conditions that are included on the current PGT-P panel. The couple also reported three first trimester miscarriages with a normal karyotype. The couple denied a history of parental chromosome rearrangements and previous pregnancies or family history of aneuploidy. The couple declined a family history of additional genetic conditions that they wished to test for via PGT studies.

### 2.2. PGT-P Case Series including Unknown Family History

To begin to evaluate the frequency of high-risk embryos across different degrees of family history, 24 consecutive PGT cases were analyzed and compared. PGT was performed using trophectoderm-biopsy-derived DNA, followed by whole-genome amplification and Axiom UKBB SNP-array-based analyses as previously described [10]. For each case, parental DNA was analyzed, and the ethnicity was predicted (Caucasian, Asian, African, other) with a pipeline built on a previously established supervised admixture methodology [13] and trained with 551 known ancestry samples [14]. The internal validation was performed on 229 samples from Coriell Cell Repository [15], resulting in an accuracy of 99.6% (228/229).

This consecutive PGT case series included couples who consented to research during genetic counseling for routine clinical use of PGT. Indications ranged from unknown family history to having 1st-degree relatives (i.e., the embryo’s sibling or parent) affected with a polygenic disease. In all cases, PGT-A was performed in parallel and from the same biopsy, as previously described [10]. Risk of type 1 and 2 diabetes, breast, prostate, and testicular cancer, malignant melanoma, basal cell carcinoma, heart attack, coronary artery disease, hypercholesterolemia, and hypertension was tested in embryos with Caucasian ancestry, and risk of type 2 diabetes, hypercholesterolemia, and hypertension in embryos with Asian ancestry. High risk of polygenic disease was defined as previously described [12].

### 2.3. PGT-P in 11,883 Adult Sibling Pairs

A recent study reported that SNPs which are predictive of specific diseases do not overlap with one another [16]. This suggests that genetic selection to avoid one disease may not result in increasing another (pleiotropy). Instead, there may exist a positive effect of combining predictors into one “index” score. A genomic index algorithm, Equation (1), was developed by combining *P_i_* (the absolute probability of getting the disease computed from SNP genotypes) with quality-adjusted life year QALY weights [17] determined by *Q_i_* (the effect on life expectancy from each disease measured as lifespan impact years) and *PA_i_* (the population average probability of getting the disease):

(1) $$\mathrm{Gi}=\sum_{i}\left(Qi\left(\mathrm{PA}i-Pi\right)\right),$$

where *i* extends over all of the disease predictors, including type 1 and 2 diabetes, breast, prostate, and testicular cancer, malignant melanoma, basal cell carcinoma, heart attack, coronary artery disease, high cholesterol, and hypertension [18]. The genomic index = Gi is the sum of each of these contributions. Life expectancy effects Q_i_ are sourced from the medical literature [19,20,21,22,23,24,25].

Predictors were constructed from data obtained from the UK Biobank by first selecting the top 50,000 SNPs (by *p*-value) obtained from GWAS generated using the PLINK software (version 1.9, Cognitive Genomics Lab, Shenzhen, China) and then using the LASSO-path algorithm from the Python Scikit Learn package [26]. The UK Biobank identified all pairwise relationships stronger than 2nd cousins using the King kinship software. These results were used to identify all individuals who were within a sibling pair. This set of sibling pairs was further restricted to all individuals who self-reported their ethnic background to be "White, British, Irish or Any Other White Background" and was set aside as a final testing set [27]. The remaining non-sibling-paired self-reported white individuals were used as a training cohort. A small set of 500 cases/controls were withheld from the training cohort to tune the LASSO hyperparameter and select the final model—the value chosen is such that the AUC between cases/controls was maximized.

In order to validate the application of the genomic index to real sibling data, and to address the potential impact of pleiotropy upon PGT-P, a genomic index score was generated for same-sex sibling pairs from the genome-wide genotyping data of the UK Biobank [27]. In each pair, one of the two siblings was assigned to the cohort of “higher-risk sibling” (worse index score sibling), and one to “lower-risk sibling” (better index score sibling). Then, the prevalence of disease was calculated among the two cohorts. The prevalence of disease in the lower-risk sibling selected cohort was compared to the randomly selected cohort using binomial testing. Sex-specific relative risk reductions for diseases which affect both sexes were averaged. 

Finally, genomic indexing was tested on blastocysts from the consecutive case series cohort described in Section 2.2, and with respect to the extent of family history of polygenic disease. Family history of polygenic disease was divided into three main categories with respect to the tested embryos: (1) having one or more first-degree affected relatives, for example an affected parent or an existing affected sibling; (2) having one or more second-degree or higher affected relatives, such as a grandparent or cousin; and (3) unknown or not reported by the patient. A two-tailed pairwise t-test was computed to compare the average genomic index of embryos among the three family history categories. 

## 3. Results

### 3.1. PGT-P Case with First-Degree Affected Family History

**Figure 2:**
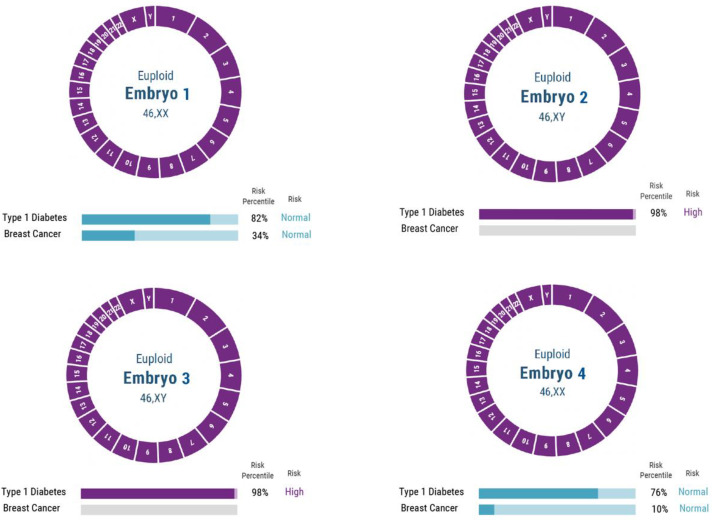
Type 1 diabetes case PGT-P results. Risk percentile indicates the predicted risk in terms of the computed polygenic risk score with respect to the distribution of risk scores from the UK Biobank cohort. Risk is classified as high when the embryo polygenic risk score is in the top 2% when compared to the average population-matched sample; otherwise, it is classified as normal risk.

Four euploid embryos were evaluated for polygenic disease risk and this resulted in identification of two at high risk for T1D (Figure 2). 

### 3.2. PGT-P Case Series Including Unknown Family History

Based upon these results, involving a case where the embryos had a first-degree relative affected by a polygenic disease, and prior results where embryos had a more distant relative (second-degree relative or higher) affected by a polygenic disease [12], we investigated the potential for correlation between the frequency of high-risk embryos produced and the extent of an embryo’s family history of polygenic disease in a larger cohort of cases. A consecutive series of 24 PGT cases with 181 embryos was evaluated by PGT-P analysis. The mean maternal age was 34.5. Thirty-seven percent of the embryos were aneuploid (67/181). Ten couples were predicted as Asian and 14 as Caucasian. There were no high-risk embryos identified from the Asian euploid embryo cohort (0/28 with no known history, and 0/3 with a more distant affected relative). Among Caucasian cases, 3 out of 51 euploid embryos (6%) were identified as high risk from couples with no known or reported family history, 3 out of 28 (11%) in cases with a more distant relative, and 4 out of 4 (100%) in a case with a first-degree affected relative. 

### 3.3. Genomic Index Selection in 11,883 Adult Sibling Pairs

**Figure 3:**
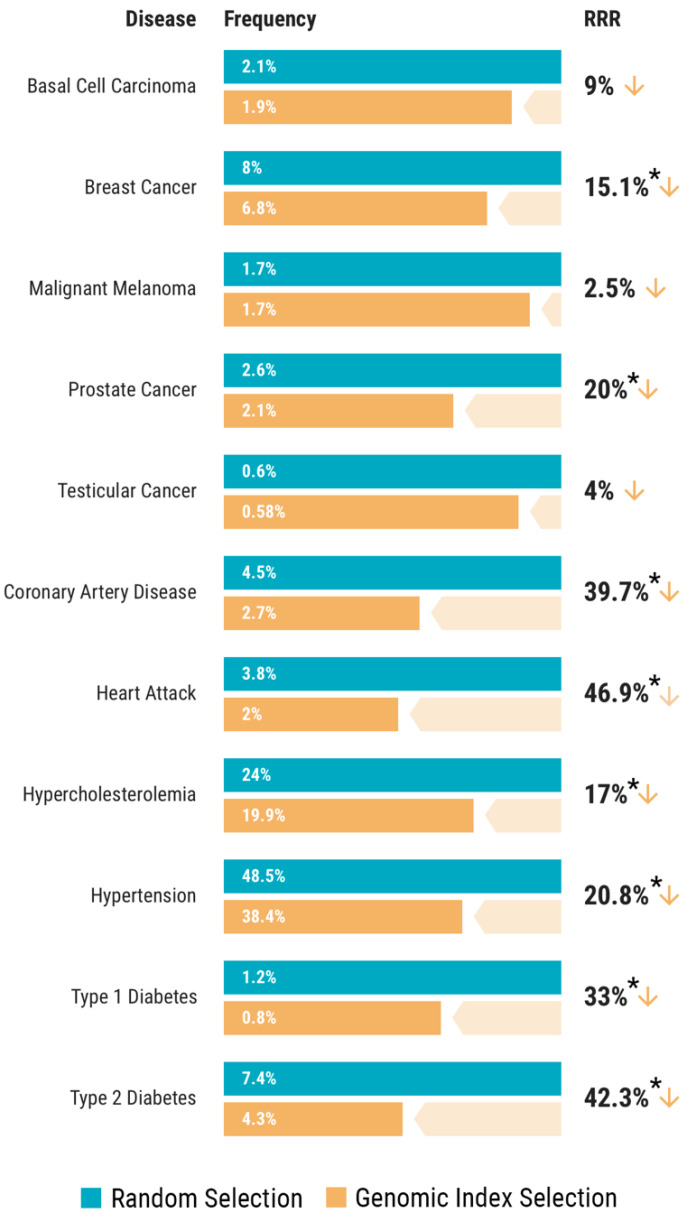
Relative risk reduction (RRR) across 11 diseases using genomic index selection compared to random selection within 11,883 sibling pairs. The frequency of disease with random selection is shown in blue, while the frequency of disease with genomic index selection is shown in orange. These data show a clear benefit from genetic selection of one of only two siblings with an unknown family history of disease. * *p*-value < 0.05 (Table 1).

A cohort of 11,883 sibling pairs was available for analysis from the UK Biobank. The intent of evaluating genomic indexing in this cohort was to model application of PGT-P in families with no known history of disease. However, the prevalence of disease in this cohort was often lower than what has been reported for the general population, which would bias the results of PGT-P in the direction of finding no reduction in risk. For instance, the prevalence of breast cancer in this UK Biobank cohort was 8.0%, while it has been reported as a 12.3% lifetime risk in the general population [28]. Likewise, 7.4% of individuals in the UK Biobank adult sibling cohort were affected with type 2 diabetes, whereas a prevalence of 9.8% has been estimated in the United States [29]. Nonetheless, these sibling pairs were used to compare the relative risk of disease with either random selection or blinded genetic selection of one of the two siblings. Results indicate a relative risk reduction for all diseases tested (Figure 3) (Table 1).

**Figure 4:**
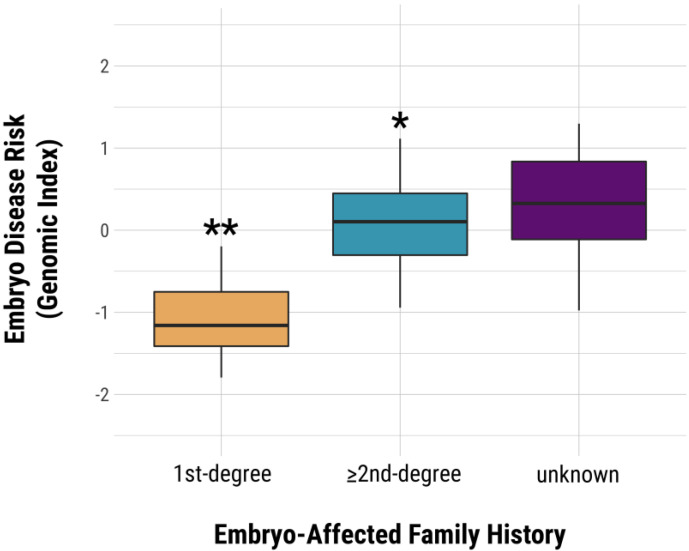
Preimplantation embryo genomic index versus family history. Embryos with a 1st degree affected relative have a significantly higher risk of polygenic disease than embryos with an unknown family history of polygenic disease. ** *p* = 0.0015 vs unknown. * *p* = 0.0129 vs unknown.

Genomic indexing was also performed on embryos evaluated in the PGT-P case series described in Section 3.2. Each embryo was classified based on the aforementioned categories of family history Results indicate that embryos with a first-degree affected relative have a higher genomic risk index compared to embryos with a more distant affected relative (*p* = 0.0132, *t* = 3.09, *df* = 9, *a* = 0.05) or with unknown family history (*p* = 0.0015, *t* = 4.34, *df* = 10, *a* = 0.05). Likewise, even embryos with at least one distant affected relative presented a higher average genomic index compared to those with unknown family history (*p* = 0.0129, *t* = 2.55, *df* = 66, *a* = 0.05) (Figure 4).

## 4. Discussion

This study extends the validity of PGT-P to reduce disease risk beyond families with a known history of disease. While many patients may elect to utilize PGT-P specifically because of a personal or family history of disease, the data presented here demonstrates utility in a more general application to routine embryo selection. One unique feature of this method is that PGT-A results are obtained in parallel with PGT-P [10], allowing patients to elect for additional information after knowing how many euploid embryos are suitable for transfer. In other words, instead of choosing which embryo to transfer based on morphology, choosing based upon PGT-P provides an option for patients to reduce the risk of polygenic disease, even when only two euploid embryos are available to choose from and when the intended parents have no known family history of polygenic disease.

In the case series reported here, 114 of 181 embryos tested were chromosomally normal (63%). Among the euploid embryos, only ten (5%) were identified as having a high risk of a polygenic disease. With this information, patients would still be faced with deciding which euploid normal risk embryo to choose for embryo transfer. Additional empirical analyses with the use of genomic indexing demonstrated relative risk reduction in all diseases tested, thereby providing additional criteria for patients to choose which embryo to transfer. Again, risk reduction was demonstrated with only two siblings to select from. Based upon a previous study, the availability of more than two siblings will further improve the relative risk reductions observed here [12].

Another important consideration relates to the potential for pleiotropy, the genetic effect of a single gene on multiple phenotypic traits [30]. With respect to PGT-P, avoiding high risk of one disease may lead to increased risk of another. The present study also demonstrated that negative pleiotropy was not observed. That is, selection with PGT-P resulted in a reduction in risk for all diseases in parallel. In support of this observation, a recent study [16] reported that SNP sets used to predict the risk of different diseases were largely disjoint. 

Although the present study clearly demonstrates the utility of PGT-P-based sibling selection to reduce the relative risk of disease, several improvements may still be possible. The current metric of the impact of each disease used in the genomic index was limited to reported years of lost life. Several studies on the burden of disease have incorporated more comprehensive metrics, including reduced quality of life [6,31,32]. More validation can be performed and optimized on the genomic index by testing it on the life span and quality-of-life outcome data from the UKBB. In addition, patients may have unique interests in reducing the risk of certain diseases over others. More careful curation of these metrics will likely improve the utility of PGT-P. 

While the clinical utility of tracing monogenic disorders through detailed pedigree analysis is well established [33], family history alone has been shown to be less effective as a single predictor of polygenic disease [34]. The results presented here may also have implications similar to when expanded carrier screening was introduced to contemporary genetic testing strategies [35]. Just as ethnicity and family history cannot be completely relied on to identify couples at risk for recessive disease, family history and ethnicity cannot be relied on alone to predict polygenic risk. That is, there is clear benefit to PGT-P in situations where “no known family history” exists, given that this status may only indicate that there was no reported history or no confirmed history, and that most families have a relative with at least one of the polygenic diseases tested by PGT-P [6,7]. This also may further benefit couples who have no known history because they know very little about their family tree, were adopted, or are using gamete donation.

## 5. Conclusions

In conclusion, PGT-P provides an additional method for embryo selection beyond conventional aneuploidy screening and morphological assessment and is applicable to prospective parents whose embryos have family histories ranging from an affected first-degree relative to no known history. At each level of embryonic family history evaluated, and in consideration of reducing the risk of polygenic disease through selection, this study demonstrates a measurable reduction in disease risk. Future work will involve incorporating additional quality-of-life metrics and DNA repository datasets, additional disease predictors, analysis of correlation with embryonic morphological characteristics, and relative risk reduction with more than two siblings to select from. The ability of genomic indexing to reduce risk of multiple diseases in parallel may allow an indirect reduction in risk of diseases where direct genomic predictors are not yet available.

## Figures and Tables

**Table 1:** Binomial test *p*-values for relative disease risk reduction between random selection and genomic index selection of 11,883 sibling pairs.

Disease.	Male	Female
Basal Cell Carcinoma	0.0224	0.2655
Breast Cancer		0.0001
Malignant Melanoma	0.3518	0.4661
Prostate Cancer	0.0224	
Testicular Cancer	0.5	
Coronary Artery Disease	9.53 × 10^−16^	3.09 × 10^−7^
Heart Attack	7.31 × 10^−22^	1.24 × 10^−6^
Hypercholesterolemia	4.73 × 10^−10^	1.21 × 10^−11^
Hypertension	3.03 × 10^−25^	3.08 × 10^−33^
Type 1 Diabetes	0.0019	0.0083
Type 2 Diabetes	1.64 × 10^−17^	2.09 × 10^−21^
